# Novel Approaches for Inhibiting the Indoor Allergen Der f 2 Excreted from House Dust Mites by Todomatsu Oil Produced from Woodland Residues

**DOI:** 10.3390/ijerph191710881

**Published:** 2022-08-31

**Authors:** Yichun Lin, Christian Ebere Enyoh, Qingyue Wang, Senlin Lu, Wei Zhang, Kai Xiao, Shumin Zhou, Toshihiko Kaneko, Akifumi Seguchi, Weiqian Wang, Yue Guo

**Affiliations:** 1 Graduate School of Science and Engineering, Saitama University, 255 Shimo-Okubo, Sakura-ku, Saitama 338-8570, Japan; 2 School of Environmental and Chemical Engineering, Shanghai University, 99 Shangdalu, Baoshan District, Shanghai 200444, China; 3 School of Life Sciences, Shanghai University, 99 Shangdalu, Baoshan District, Shanghai 200444, China; 4 Japan Aroma Laboratory Co., Ltd. (S. T. Corporation), Tokyo 161-8540, Japan

**Keywords:** avoidance strategy, todomatsu oil, woodland residues, house dust mite (HDM), allergen Der f 2, allergenicity, molecular docking

## Abstract

House dust mite (HDM) is a globally ubiquitous domestic cause of allergic diseases. There is a pressing demand to discover efficient, harmless, and eco-friendly natural extracts to inhibit HDM allergens that are more likely to trigger allergies and challenging to be prevented entirely. This study, therefore, is aimed at assessing the inhibition of the allergenicity of major HDM allergen Der f 2 by todomatsu oil extracted from residues of *Abies Sachalinensis*. The inhibition was investigated experimentally (using enzyme-linked immunosorbent assay (ELISA), surface plasmon resonance (SPR), and sodium dodecyl sulfate-polyacrylamide gel electrophoresis (SDS-PAGE)) and in silico using molecular docking. The results showed that todomatsu oil inhibits the allergenicity of Der f 2 by reducing its amount instead of the IgG binding capacity of a single protein. Moreover, the compounds in todomatsu oil bind to Der f 2 via alkyl hydrophobic interactions. Notably, most compounds interact with the hydrophobic amino acids of Der f 2, and seven substances interact with CYS27. Contrarily, the principal compounds fail to attach to the amino acids forming the IgG epitope in Der f 2. Interestingly, chemical components with the lowest relative percentages in todomatsu oil show high-affinity values on Der f 2, especially β-maaliene (−8.0 kcal/mol). In conclusion, todomatsu oil has been proven in vitro as a potential effective public health strategy to inhibit the allergenicity of Der f 2.

## 1. Introduction

Allergenicity refers to the ability of an antigen to elicit an aberrant immune response, which is an overreaction that is distinct from a normal immune response in that it does not result in a protective/prophylaxis effect but rather causes physiological function disturbance or tissue damage [1]. Allergies have been more common during the last few decades all around the world [2]. House dust mite (HDM) is the most widespread domestic allergen source and a crucial factor in allergic illnesses such as allergic rhinitis, allergic asthma, and atopic dermatitis [2,3,4]. Consequently, preventing allergy to house dust mites is an urgent public health concern.

There are 22 HDM allergens identified, with group 1 and group 2 allergens of *Dermatophagoides pteronyssinus* (Der p 1 and Der p 2) and *Dermatophagoides farinae* (Der f 1 and Der f 2) being the most dominant [2,5,6], with over 80% of patients developing high levels of specific IgE antibodies to these allergens [7]. Group 2 mite allergens is a category of MD2-related lipid-recognition proteins [8,9]. Among them, one of the primary mite allergens Der f 2 is abundant in the intestinal tract and feces, from where it is carried to the lungs by mite airborne fecal particles [10,11,12].

HDM allergens can induce allergy by triggering a Th2-biased adaptive immune response [2,5,6,7,13]. Simply, HDM allergens are inhaled to reach the airways and, thus, the mucosa. Once the inhaled allergens reach the bloodstream of the mucosa, they are presented by myeloid dendritic cells (mDC) to T cells and recognized by T cells via the T cell receptor (TCR). As a result, T cells differentiate into Th2 cells. The HDM-induced allergic response is then orchestrated by activated Th2 cells, which stimulate the synthesis of HDM allergen-specific IgE and the recruitment of inflammatory cells, resulting in structural alterations in the lungs, nose, and skin. For Der f 2, composed of 129 amino acids with a molecular weight of around 15 kDa, the conformational integrity of the protein, rather than the continuous sequence of amino acids, is required for group 2 allergens recognition by its particular antibody [10,11,14]. It was proven that the existence of three disulfide bonds (CYS8-CYS119, CYS21-CYS27, and CYS73-CYS78) is critical in maintaining the stable tertiary structure linked to allergenic characteristics [15].

House dust mites have been removed from our living environment using a variety of approaches, including high-temperature steam cleaning, microfibers, air ionizer, high-efficiency particulate air filtration (HEPA) vacuum cleaners, air purification, humidity control (below 50%), and chemical synthetic agent sterilizing [16,17,18,19,20,21,22,23,24,25,26,27,28,29]. Phthalthrin cyclopropanecarboxylate, benzyl benzoate, and N,N-diethyl-m-toluamide [30] are the major chemical components of regularly used insecticides. It is worth noting that while these substances help eliminate house dust mites, they develop resistance when used repeatedly and may disrupt the ozone layer in the atmosphere, posing a risk to the environment and human health [31]. Additionally, the allergens in the fecal particles excreted by HDMs are more likely to elicit allergies and are difficult to avoid entirely. As a result, natural acaricides of ecologically benign natural components that are safe for humans and have strong removal characteristics to substitute synthetic agents are urgently needed. As it becomes clear that plant-derived acaricides have immense potential in this area, research into them is being stepped up. Essential oils produced from natural products have long been utilized in perfumery, culinary smells, and aromatherapy because they contain insecticidal components known as monoterpenes [32,33,34]. Many essential oils with antioxidant capacity and antimicrobial activity had been found, such as extracts from *Lavandula angustifolia* and *Cinnamomum camphora chvar. Borneol* [34,35]. The prior reports showed that HDMs could be effectively controlled by essential oils produced from plants such as *Theobroma cacao L.*, *Salvia lavandulifolia*, and *Cinnamomum camphora chvar. Borneol* [16,33,34]. Furthermore, the essential oils from medicinal plants such as cinnamon (*Cinnamomum bejolghota* [Buch.-Ham.] Sweet), citronella grass (*Cymbopogon nardus* Rendle), and clove (*Syzygium aromaticum* [L.] Merr. & L. M. Perry) decrease the allergen Der p 1 from HDMs [36]. Nevertheless, investigation regarding the suppression of allergen Der f 2 is far behind.

Todomatsu oil, extracted from woodland residues such as wood, bark parts, and leaves in Hokkaido, Japan, is one of the most popular commercialized products that can decrease the cedar pollen allergenicity [37]. The forest area in Japan accounts for about 68% of its country’s land area, and Japan is one of the few countries in the world with a high forest coverage rate. Therefore, the necessary technologies to utilize wood waste resources to reduce the burden on the environment are vigorously developed in Japan. The exploitation of todomatsu oil, derived from residues of *Abies sachalinensis* like bark parts and leaves which have not yet been effectively utilized, is low-cost, can be used in large quantities, and is beneficial to the sustainable development of the environment from the standpoint of environmental conservation. Besides, it is the first time to create essential oil from plant waste to reduce allergies. The active ingredients of todomatsu oil from *Abies sachalinensis* (including 3-carene, α-terpinolene, borneol, β-maaliene, tricyclene, β-myrcene, limonene, β-pinene, β-phellandrene, α-pinene, camphene, and bornyl acetate [37]) coat cedar pollen and reduce the antibody binding reactivity of its allergens to about 10% [38]. The immune pathogenesis of pollinosis to trigger Th2-associated immune response is similar to house dust mite allergy. Therefore, the question about the capability of todomatsu oil on inhibiting HDM allergenicity was raised.

This study evaluated the in vitro inhibition of todomatsu oil from the residues of *Abies Sachalinensis* on the allergenicity of allergen Der f 2, one of the major allergens of HDM, according to the variation in the content of Der f 2 and IgG binding ability of a single allergen. Since serum only contains trace levels of natural IgE antibodies, the approach described here selected an IgG antibody, anti-Der f 2 monoclonal antibody (mAb) 15E11, as a surrogate for IgE. The mAb 15E11 epitope overlaps with human IgE epitopes [39]. In addition, molecular docking, docking small-molecule libraries to a macromolecule to uncover lead compounds with the specified biological function, is well known for its extensive application in computer-aided drug design [40]. The main results from virtual molecular docking are the binding modes and corresponding binding affinities that show the binding abilities of the compounds on macromolecules [40,41]. Thus, the binding sites and affinities of chemicals in todomatsu oil on Der f 2 were further examined by molecular docking in silico to explain how todomatsu oil affects Der f 2 and to assess their abilities to bind to Der f 2, respectively. These findings shed light on how HDM allergen Der f 2 may be managed in the environment by harmless, low-cost, and ecologically friendly plant extracts and how to avoid house dust mite allergy.

## 2. Materials and Methods

### 2.1. Pretreatment of Samples by Todomatsu Oil

**Figure A1:**
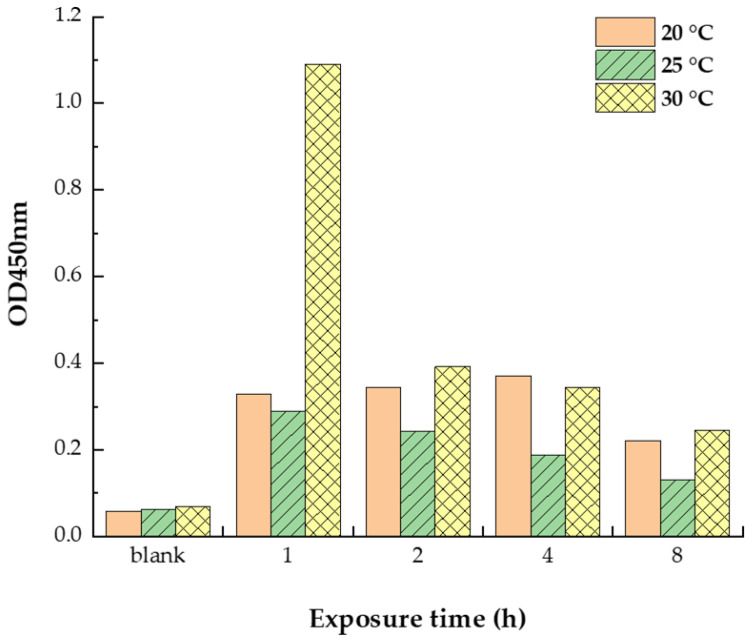
The effect of temperature on allergen Der f 2. Der f 2 (250 ng/mL, 250 ng/mL, and 2000 ng/mL, at 20 °C, 25 °C, and 30 °C, separately) were treated for 1, 2, 4, and 8 h. The results represent the mean of three independent replicates. −/+: Samples of Der f 2 without/with todomatsu oil.

Todomatsu oil (Aroma Laboratory Co., Ltd., Shinjuku, Tokyo, Japan) treatment was conducted in a 200-mL airtight vessel. The pretreatment of samples of the allergen Der f 2 (Shibayagi Co., Ltd., Shibukawa, Gunma, Japan) for enzyme-linked immunosorbent assay (ELISA), surface plasmon resonance (SPR) experiments, and sodium dodecyl sulfate-polyacrylamide gel electrophoresis (SDS-PAGE) are shown in Table 1. For ELISA, the allergens Der f 2 (2000 ng/mL, 250 ng/mL, and 250 ng/mL at 30 °C, 25 °C, and 20 °C, separately) were treated with evaporated todomatsu oil (0.05 μL/cm^3^) for 1, 2, 4, and 8 h, at 30 °C, 25 °C, and 20 °C (natural room temperatures in summer and air conditioning room temperature in summer and winter). The concentrations of Der f 2 were determined by the impact of temperature on Der f 2 (shown in Figure A1 in Appendix A). The allergen Der f 2 was diluted by PBS (pH7.4). For SPR experiments, Der f 2 was diluted by HBS-EP buffer (general purpose buffer, Cytiva Co., Ltd., Shinjuku, Tokyo, Japan) with 5 concentrations (500, 250, 125, 62.5, and 31.25 ng/mL) and were treated with todomatsu oil (0.05 μL/cm^3^) at 25 °C for 1 h. For SDS-PAGE, Der f 2 (100 μg/mL) was treated with todomatsu oil (0.05 μL/cm^3^) at 25 °C for 2 h. The allergen Der f 2 treatment without todomatsu oil was also performed in the same way as the control in each experiment.

### 2.2. Der f 2 Allergenicity Identification via Enzyme-Linked Immunosorbent Assay (ELISA)

Allergenicity of allergen Der f 2 with and without todomatsu oil was assessed employing ELISA [42,43,44]. The pretreatments of samples are shown in Table 1. Polystyrene plates were coated with 100 μL/well of allergen samples, which are the allergens treated above, dissolved in carbonate buffer (pH 9.6) (1:1 *v*/*v*) and incubated overnight at 4 °C, avoiding light. The wells were washed with PBST (PBS with 0.1% Tween-20 *v*/*v*) 3 times and blocked with 100 mg/L Bovine serum albumin (BSA, Jackson ImmunoResearch Laboratories Inc., West Grove, PA, USA) in PBST (300 μL/well) for 1.5 h at 37 °C. Plates were incubated for 2 h at 37 °C with mouse IgG1 mAb anti-Der f 2 (15E11, Shibayagi Co., Ltd., Shibukawa, Gunma, Japan) (100 μL/well) and repeatedly washed as before. Then the plates were incubated for 1.5 h at 37 °C with goat HRP-conjugated anti-mouse IgG polyclonal secondary antibody (R&D Systems) (100 μL/well). The color development was carried out with TMB (3,3′,5,5′-tetramethylbenzidine) solution (Thermo Scientific Inc., Waltham, MA, USA) (100 μL) at 37 °C for 30 min after washing 5 times and was stopped with 100 μL of 2 M H_2_SO_4_. Finally, the absorbance value of the samples was detected by a microplate reader (Bio-Rad Laboratories, Inc., Hercules, CA, USA) at the wavelength of 450 nm (OD450). The following blank control was used: wells coated with PBS (instead of samples) and incubated with 5% BSA, mouse IgG1 mAb anti-Der f 2, and goat HRP-conjugated anti-mouse IgG polyclonal secondary antibody following the process described above. All the tests were performed in triplicate. The inhibition rates were analyzed with the mean of triplicate through the following Formula (1):

(1) $$\mathrm{Inhibition}\;\mathrm{rates}\;\left(\%\right)=\frac{\left(B_{C}-B_{T}\right)}{\left(B_{C}-B_{0}\right)}\times 100\%$$

where *B_C_*, *B_T_*, and *B*_0_ are the absorbance values of the wells of Der f 2 without todomatsu oil, Der f 2 with todomatsu oil, and blank control, respectively.

### 2.3. Der f 2 Content Determination Using Sodium Dodecyl Sulfate-Polyacrylamide Gel Electrophoresis (SDS-PAGE)

Allergen Der f 2 content from todomatsu oil treatment was analyzed by SDS-PAGE [43,45,46]. Briefly, SDS-PAGE was performed with polyacrylamide gels. Table 1 shows how samples were pretreated. 8 µL samples were mixed with 1.5 µL of 6× loading buffer (Geno Technology Inc., St. Louis, MO, USA), then denatured by water-bath heating at 99 °C for 5 min. Each sample was loaded onto the 12.5% prefabricated gels (ATTO Co., Ltd., Chuo, Tokyo, Japan) and electrophoresed at 250 constant voltages for 90 min. After electrophoresis, the gel was stained with Coomassie brilliant blue R-250 (ATTO Co., Ltd., Chuo, Tokyo, Japan) and de-stained with deionized water. The gray values of the bands were determined by Image J analysis software version 1.48 (National Institutes of Health, Bethesda, MD, USA).

### 2.4. IgG Binding Capacity of a Single Protein Der f 2 Examination through Surface Plasmon Resonance (SPR) Experiments

Surface plasmon resonance (SPR) is recognized as the gold standard for measuring binding [47,48]. SPR tests on CM5 sensor chips (Cytiva Co., Ltd., Shinjuku, Tokyo, Japan) were carried out at 25 °C using a Biacore X100 device (Cytiva Co., Ltd., Shinjuku, Tokyo, Japan). The sensorgrams of SPR were evaluated according to the manufacturer’s instructions for the Biacore X100 Evaluation software (Cytiva Co., Ltd., Shinjuku, Tokyo, Japan). Anchoring antibodies (anti-Der f 2) in 10 mM acetate buffer (pH 5.0) were immobilized by amine coupling according to the manufacturer’s instructions (Cytiva Co., Ltd., Shinjuku, Tokyo, Japan). The sensorgrams were referenced using a flow cell that was left blank. Spikes that remained after this phase were not deleted since they did not affect the results. HBS-EP running buffer was used to prepare the samples, then injected onto the functionalized surface. A 1 min pulse of 10 mM glycine pH 3.0 (Cytiva Co., Ltd., Shinjuku, Tokyo, Japan) was used to perform regeneration at the end of each kinetic cycle. The HBS-EP solution was chosen as the control.

The investigations were carried out utilizing the single-cycle kinetics (SCK) method [47,48]. Table 1 shows the pretreatment of samples. The analyte (Der f 2) was injected five times in increasing quantities, with no regeneration in between. Direct curve fitting of the sensorgrams to a Langmuir 1:1 model of interaction yielded the association (K_a_) and dissociation rate constants (K_d_), as well as the dissociation equilibrium constant (K_D_). The binding strength of the antigen and its specific antibody is represented by K_D_. A lower K_D_ value indicates a higher binding capability.

### 2.5. In Silico Binding Sites and Affinities of Compounds in Todomatsu Oil on Der f 2

**Figure 1:**
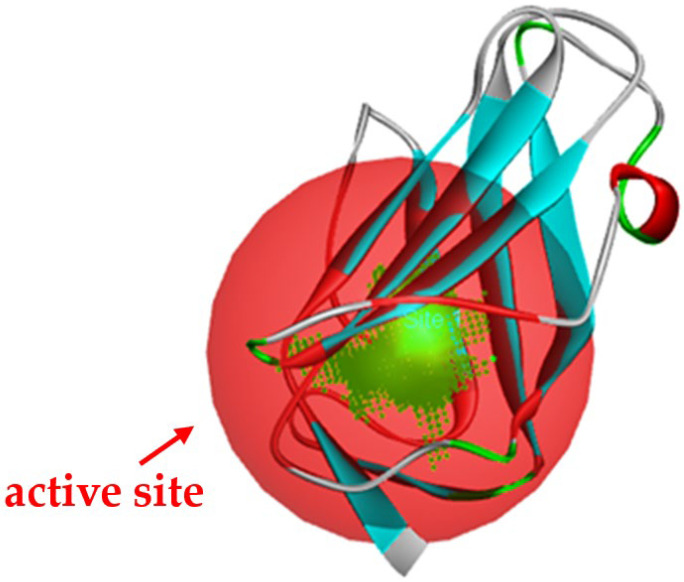
Allergen Der f 2 (PDB ID: 1WRF) of *Dermatophagoides farinae* showing active site. Active site showed by a red sphere with a green center and pointed by the red arrow.

Molecular docking in silico was applied to estimate the binding sites and affinities of the chemicals in todomatsu oil on Der f 2. Before docking analysis, the preparation of protein and ligands was achieved. The composition of todomatsu oil was provided by Forestry and Forest Products Research Institute, Wood extractives laboratory, and Japan Aroma Laboratory [37]. The docking analysis was performed using 3D structure-data files (SDF) of the compounds acquired from the PubChem database (https://pubchem.ncbi.nlm.nih.gov/) (accessed on 9 December 2021). From the Protein Data Bank (PDB) (https://www.rcsb.org/) (accessed on 9 December 2021), the allergen Der f 2 (PDB ID: 1WRF) was retrieved. AutoDock Vina was in charge of energy minimization of ligands and protein minimization [40,49,50,51]. Biovia Discovery studio 4.5 was used to discover the active spots on Der f 2 (BIOVIA 2020). The active site of the allergen Der f 2 is shown in Figure 1.

The multiple ligand docking of the main compounds in todomatsu oil on the Der f 2 was done with AutoDock Vina in PyRx software version 0.8 (San Diego, CA, USA) [40,52,53]. In brief, the compounds were docked blindly at the Der f 2 cavities to allow the ligands unrestricted access to engaging with sites with the lowest energy. Center x: 6.6110, center y: −1.4810, center z: 19.2795, and dimensions (Angstrom) x: 46.0495, dimensions (Angstrom) y: 48.4634, dimensions (Angstrom) z: 34.9154 were specified for the center grid box. The optimal binding modes and related binding affinities are the cardinal findings of molecular docking [40]. Biovia Discovery studio 4.5 was used to visualize hydrogen bonding and other hydrophobic interactions between the protein–ligand complex of the compounds. The binding affinities were obtained in terms of binding free energy (ΔG) for each compound. The binding between a ligand and its target macromolecule is more effective when the values of ΔG energy are more negative [40].

### 2.6. Statistical Analysis

All experiments were performed in triplicate and the data obtained were analyzed by one-way analysis of variance (One-Way ANOVA) using SPSS for windows version 18 (SPSS Inc., Chicago, IL, USA). Statistical significance was set at *p* < 0.05.

## 3. Results

### 3.1. Reduced Allergen Der f 2 Caused by Todomatsu Oil

**Figure 2:**
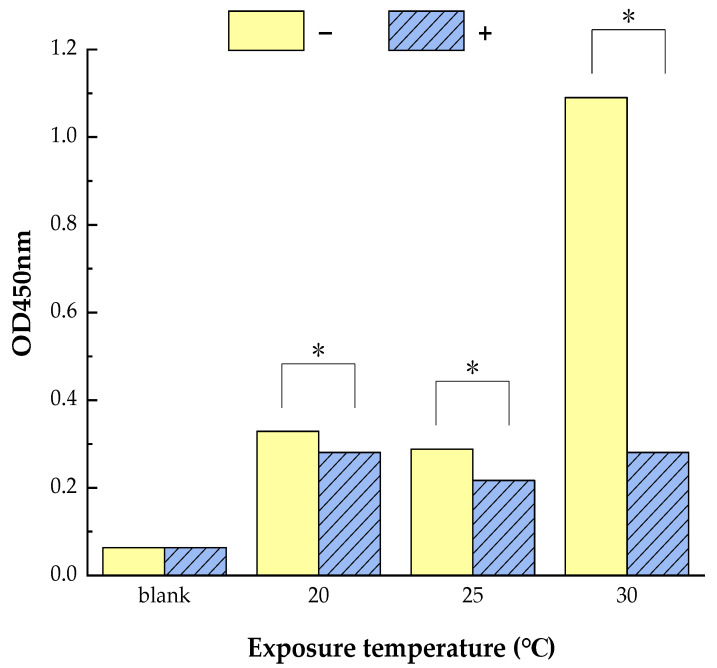
Diminished allergenicity of allergen Der f 2 was analyzed by ELISA. Der f 2 (250 ng/mL, 250 ng/mL and 2000 ng/mL, at 20 °C, 25 °C, and 30 °C, separately) were treated with and without todomatsu oil (0.05 µL/cm^3^) for 1 h. The results represent the mean of three independent replicates. −/+: Samples of Der f 2 without/with todomatsu oil. * indicates a significant difference (*p* < 0.05).

The inhibition of todomatsu oil on the allergenicity of Der f 2, one kind of main allergic protein of HDM, was analyzed by ELISA. As shown in Figure 2, the amount of allergen Der f 2 binding to IgG was significantly reduced by todomatsu oil compared to samples without todomatsu oil, no matter at 20 °C, 25 °C, and 30 °C. The results indicate that todomatsu oil inhibits the allergenicity of Der f 2.

**Figure 3:**
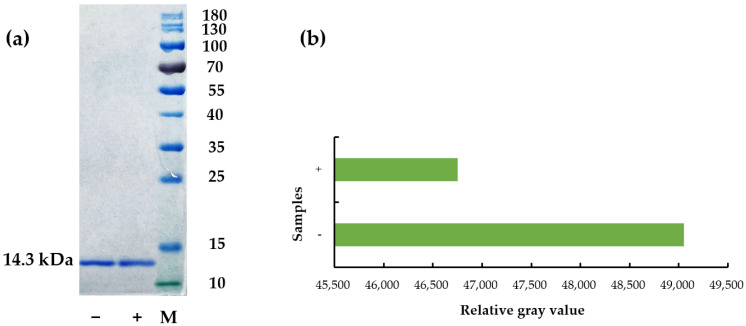
Decreased content of Der f 2 treatment with todomatsu oil was examined via SDS-PAGE. (**a**) analysis of allergen content by SDS-PAGE; (**b**) quantification of results from SDS-PAGE. Der f 2 (100 µg/mL) was treated with and without todomatsu oil (0.05 µL/cm^3^) at 25 °C for 2 h. −/+: Samples of Der f 2 without/with todomatsu oil; M: marker.

Insung et al. (2016) showed similar results that the essential oils from cinnamon (*Cinnamomum bejolghota* [Buch.-Ham.] Sweet), citronella grass (*Cymbopogon nardus* Rendle), and clove (*Syzygium aromaticum* [L.] Merr. & L. M. Perry) lowered allergenic properties of Der p 1, one of the key allergenic proteins in *Dermatophagoides pteronyssinus*, and suggested that it results from reduced allergen levels [36]. Thus, the impact of todomatsu oil on allergen amount was examined using SDS-PAGE. As shown in Figure 3, the content of allergen Der f 2 was significantly decreased compared to the sample without todomatsu oil. This suggests that todomatsu oil decreases the allergen amount, which is responsible for the ELISA results. Therefore, todomatsu oil inhibits the allergenicity of Der f 2 by lowering its amount.

**Figure 4:**
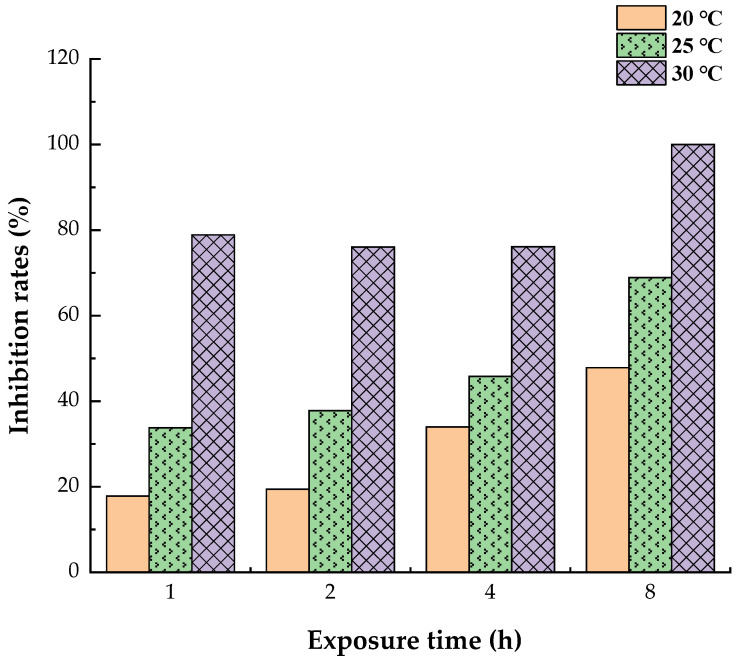
Inhibition rates of todomatsu oil on allergen Der f 2 were determined by ELISA. Der f 2 (250 ng/mL, 250 ng/mL and 2000 ng/mL, at 20 °C, 25 °C, and 30 °C, separately) were treated with and without todomatsu oil (0.05 µL/cm^3^) for 1, 2, 4 and 8 h. Three independent replicates. Inhibition rates were calculated with the mean of three independent replicates through Formula (1).

Furthermore, the inhibition rates of allergen Der f 2 by todomatsu oil were estimated. As shown in Figure 4, the inhibition rates are 17.81%, 19.41%, 33.97%, and 47.85% at 20 °C for 1, 2, 4, and 8 h; 33.78%, 37.78%, 45.82%, and 68.89% at 25 °C for 1, 2, 4 and 8 h; 78.86%, 76.05%, 76.14% and 100.0% at 30 °C for 1, 2, 4 and 8 h, respectively. The maximum inhibition (100.0%) was discovered at 30 °C after 8 h. Additionally, it appears the inhibition rates of todomatsu oil on Der f 2 rises with increased exposure temperature. The increased concentration of evaporated todomatsu oil with increased exposure temperature may contribute to that.

### 3.2. Differences in IgG Binding Capacity of a Single Protein Der f 2

**Figure A2:**
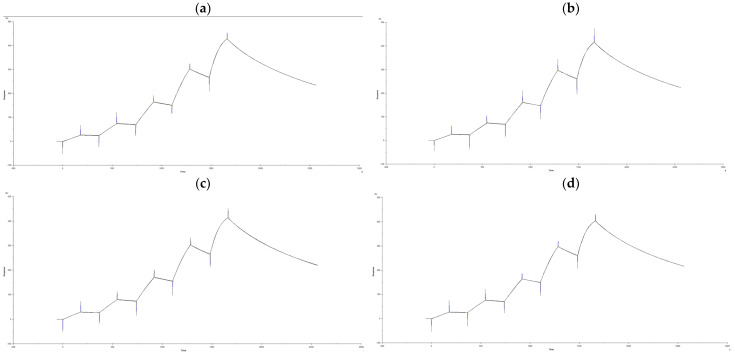
Sensorgrams of SCK about Der f 2 without (**a**,**c**) and with (**b**,**d**) todomatsu oil. Der f 2 (500, 250, 125, 62.5 and 31.25 ng/mL, at 20 °C, 25 °C and 30 °C, separately) were treated with and without todomatsu oil (0.05 µL/cm^3^) for 1 h. The single-cycle kinetics (SCK) method of Surface plasmon resonance (SPR) was performed to determine the Ig G binding ability of a single Der f 2. Two independent replicates.

The effect of todomatsu oil on the IgG binding capacity of a single protein was analyzed by SCK to determine whether todomatsu oil also inhibits allergenicity of Der f 2 by lowering the IgG binding ability of a single protein Der f 2. The sensorgrams are presented in Figure A2a–d in Appendix A. As reported in Table 2, the K_D_ values of Der f 2 with and without todomatsu oil are comparable in both replicates. It implies that the IgG binding ability of a single allergen Der f 2 remains unaffected by todomatsu oil. In contrast, the maximum binding (Rmax) of Der f 2 in samples with todomatsu oil decreased compared to the Rmax of protein Der f 2 in samples without todomatsu oil. The lowered allergen content is perhaps accountable for it.

### 3.3. The Alkyl Hydrophobic Interactions between Todomatsu Oil and Der f 2

**Figure 5:**
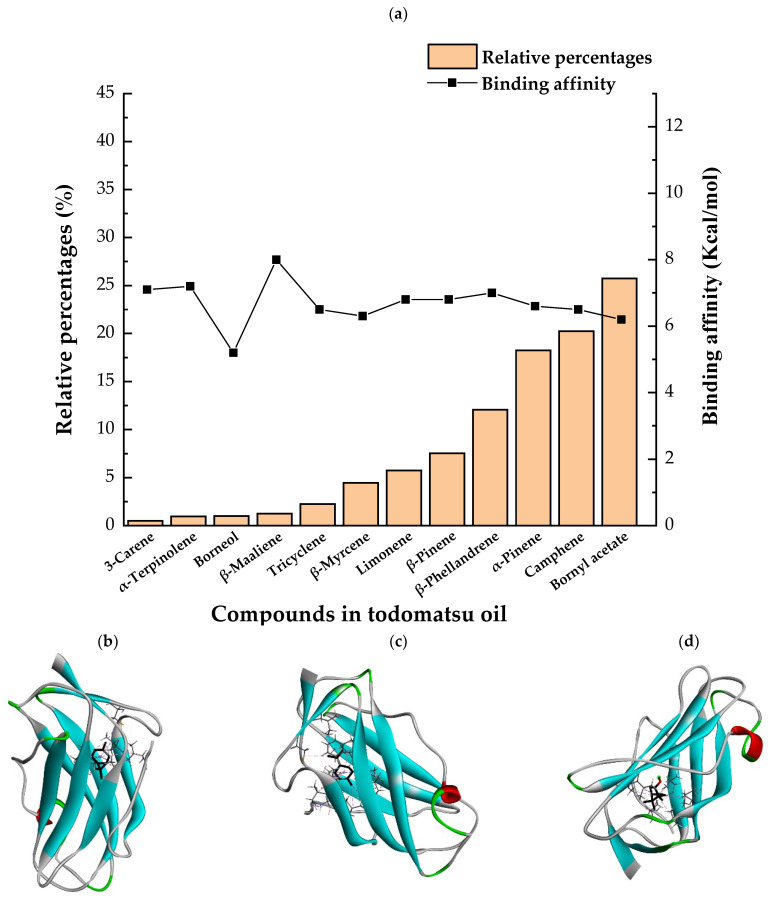

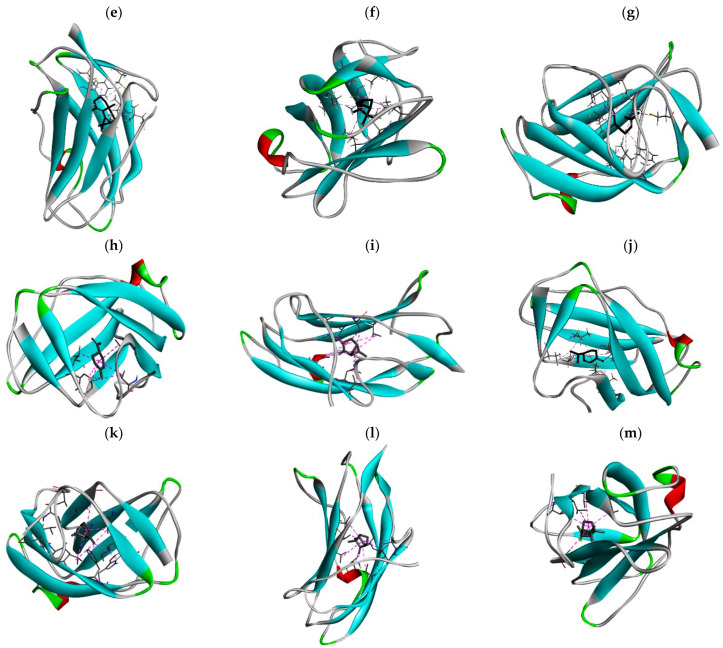
The composition of todomatsu oil (**a**), binding affinity (**a**), and sites (**b**–**m**) of the main compounds in todomatsu oil on protein Der f 2. Binding mode of 3-carene (−7.1 kcal/mol) (**b**), α-terpinolene (−7.2 kcal/mol) (**c**), borneol (−5.2 kcal/mol) (**d**), β-maaliene (−8.0 kcal/mol) (**e**), tricyclene (−6.5 kcal/mol) (**f**), β-myrcene (−6.3 kcal/mol) (**g**), limonene (−6.8 kcal/mol) (**h**), β-pinene (−6.8 kcal/mol) (**i**), β-phellandrene (−7.0 kcal/mol) (**j**), α-pinene (−6.6 kcal/mol) (**k**), camphene (−6.5 kcal/mol) (**l**), and bornyl acetate (−6.2 kcal/mol) (**m**) on Der f 2 (PDB ID: 1WRF). The composition of todomatsu oil was provided by the Forestry and Forest Products Research Institute, Wood extractives laboratory, and Japan Aroma Laboratory [37]. The relative percentages of compounds in todomatsu oil are the means of relative percentages of compounds extracted from todomatsu oil by hydro-distillation and vacuum microwave-assisted steam distillation.

The binding sites and affinities of compounds in todomatsu oil (including 3-carene, α-terpinolene, borneol, β-maaliene, tricyclene, β-myrcene, limonene, β-pinene, β-phellandrene, α-pinene, camphene, and bornyl acetate) on allergen Der f 2 were investigated in silico by molecular docking. The compounds in todomatsu oil have been discovered to bind to allergen Der f 2 via alkyl hydrophobic interactions. Hydrophobic interactions are significant for the folding of proteins correctly, as well as also crucial in keeping a protein stable and biologically active because they enable the protein to have less surface area and fewer unfavorable interactions with water. The binding positions of the compounds on Der f 2 are shown in Figure 5b–m, and the interactions of these compounds with the amino acid residues on the Der f 2 are shown in Table 3.

As shown in Table 3, most of the compounds in todomatsu oil bind to the hydrophobic amino acids buried deep inside the protein Der f 2. For example, these connections have been found between α-terpinolene and 11 amino acid residues (VAL3, VAL5, VAL16, VAL18, ILE29, PHE35, LEU37, VAL106, VAL108, ALA120, and ALA122), between β-myrcene and 8 residues (VAL5, VAL18, PHE35, LEU37, VAL106, VAL108, ALA120, and ALA122), and between tricyclene and 8 residues (VAL3, VAL5, VAL16, VAL18, LEU37, VAL106, VAL108, and ALA122), etc. Thirteen hydrophobic amino acids of Der f 2 can be bound to compounds in todomatsu oil (shown in Table 3). For instance, there are 10 compounds (borneol, β-maaliene, β-myrcene, limonene, camphene, bornyl acetate, 3-carene, α-terpinolene, tricyclene, and β-phellandrene) interacting with VAL106 and ALA122, 9 compounds (borneol, β-maaliene, β-myrcene, limonene, bornyl acetate, 3-carene, α-terpinolene, tricyclene, and β-phellandrene) interacting with VAL18, and 8 compounds (borneol, β-maaliene, limonene, camphene, bornyl acetate, 3-carene, α-terpinolene, and tricyclene) interacting with VAL3, etc.

There are three disulfide bonds (CYS8-CYS119, CYS21-CYS27, and CYS73-CYS78) critical in maintaining the stable tertiary structure in Der f 2 [15]. Based on the analysis of Der f 2 binding sites of compounds in todomatsu oil in silico, β-myrcene, limonene, camphene, 3-carene, α-terpinolene, β-maaliene, and β-phellandrene have been found in alkyl hydrophobic interactions with the amino acid residue CYS27 which developed one disulfide bond with CYS21 in Der f 2 (shown in Table 3a,b,d,f,g,i,k).

According to the report from Ichikawa et al. (2005) [8] and Nishiyama et al. (1999) [39], the Der f 2 region ranging from 69 to 78 is recognized by mAb 15E11 and especially ASP69, ASN71, and HIS74. As shown in Table 3, the main compounds in todomatsu oil are invalid to bind to these amino acids. It is a reasonable explanation for the unobserved difference of IgG binding ability to a single protein Der f 2 by todomatsu oil.

### 3.4. The Binding Affinity of Compounds in Todomatsu Oil on Der f 2

In the report from Tatsuro et al. (2010), bornyl acetate, camphene, α-pinene and β-phellandrene are the dominant compounds with the highest relative percentages in todomatsu oil (shown in Figure 5a) [37], β-pinene, limonene, and β-myrcene are followed [37], while tricyclene, β-maaliene, borneol, α-terpinolene, and 3-carene are the compounds with the lowest relative percentages (less than 3%) in todomatsu oil [37]. These compounds are bound to allergen Der f 2 by alkyl hydrophobic interactions, and their binding affinities on the Der f 2 are shown in Figure 5a and Table 3. The binding affinities display the abilities of a compound to connect to the target [40,41]. The binding affinity values of bornyl acetate, camphene, α-pinene, and β-phellandrene on allergen Der f 2 are −6.2 kcal/mol, −6.5 kcal/mol, −6.6 kcal/mol, and −7.0 kcal/mol, respectively (shown in Figure 5a and Table 3i–l). Interestingly, compounds with the lowest relative percentages in todomatsu oil show high-affinity values on Der f 2 instead. As shown in Figure 5a and Table 3a–b,d, the binding affinity value of β-maaliene (−8.0 kcal/mol) on Der f 2 is the highest, followed by α-terpinolene (−7.2 kcal/mol) and 3-carene (−7.1 kcal/mol).

## 4. Discussion

Numerous studies have been undertaken to demonstrate the importance of HDM allergens exposure, especially for respiratory allergic diseases [54,55]. In contrast, although HDM was recognized as a known allergic source, little attention was attracted to the investigation of inhibiting its allergens [36]. Insung et al. (2016) [36] reported that essential oils obtained from medicinal plants cinnamon (*Cinnamomum bejolghota* [Buch.-Ham.] Sweet), citronella grass (*Cymbopogon nardus* Rendle), and clove (*Syzygium aromaticum* [L.] Merr. & L. M. Perry) reduced the amount of Der p 1 allergen. Nevertheless, the study on the inhibition of Der f 2 allergens from HDM remains unknown. We carried out this study to evaluate the inhibition of todomatsu oil, extracted from residues of *Abies Sachalinensis* with the properties of harmless, low-cost, and ecologically friendly, to remove the allergen Der f 2 from our habitat.

The majority of compounds in todomatsu oil bind to hydrophobic amino acids buried in the middle of protein Der f 2 and 13 hydrophobic amino acids of Der f 2 can be bound to substances in todomatsu oil, as demonstrated in Table 3. The hypothesis is that the hydrophobic amino acid residues of Der f 2 exposure as the result of the alkyl hydrophobic interactions of Der f 2 with compounds in todomatsu oil, cause Der f 2 denaturation and easier to be degraded, leading to the decreased content of this allergen Der f 2.

β-myrcene, limonene, camphene, 3-carene, α-terpinolene, β-maaliene, and β-phellandrene have been found to connect with the amino acid residue CYS27 by alkyl hydrophobic interactions (shown in Table 3a,b,d,f,g,i,k), which may disrupt the disulfide bond between CYS21-CYS27. It was proved that the presence of three disulfide bonds (CYS8-CYS119, CYS21-CYS27, and CYS73-CYS78) plays an essential role in maintaining the stability of the tertiary structure [15]. Therefore, the possible broken conformational integrity of allergen Der f 2 caused by the disrupted disulfide bond between CYS21-CYS27 probably accelerates the allergen degrading.

Though the remarkable conformational change produced by the breakdown of intramolecular disulfide bonds in Der f 2, Takai et al. (2000) and Yang et al. (2012) suggested that the IgE/IgG-binding capacity of Der f 2 depends on disulfide bonds, CYS8-CYS119 and CYS73-CYS78, rather than CYS21-CYS27 [14,56]. Therefore, the cleavage of the disulfide bond CYS21-CYS27 is insufficient to induce a considerable variation in the IgG binding capacity of a single allergen.

The results of this study provide a promising strategy to avoid house dust mite allergy. Even though the practical application has not been made in this article, todomatsu oil, which has been utilized as a preventative measure for cedar pollen allergies, can be applied in the same way for allergies to house dust mites. Todomatsu oil can be not only sprayed indoors but also applied as a paste on masks while going outside (house dust mites also can be present in public locations such as offices and public transportation) [57]. The bright spot of this research is that todomatsu oil originated from *Abies Sachalinensis* residues, making it low-cost and environmentally friendly, rather than the more expensive medicinal plants. In addition, the inhibition of todomatsu oil on the amount of protein Der f 2 and the IgG binding ability of a single allergen were investigated. Furthermore, the binding sites and affinities of todomatsu oil for allergen Der f 2 were analyzed in silico. These findings that help to understand how allergen Der f 2 is affected by todomatsu oil provide the orientation for future research, like the impact of a single ingredient in todomatsu oil on allergens of house dust mites. Besides, extracting methods to increase the efficient composition of todomatsu oil should be improved. Jakobsen et al. (2005) proposed comparing the binding affinities of IgE, IgG1, and IgG4 to the dominant allergen Bet v 1 of birch pollen via SPR [58]. It provides an idea for our future research, suggesting it will be more accurate to predict the inhibition of todomatsu oil on the human IgE binding to Der f 2 if the binding affinity of human IgE and mAb IgG to Der f 2 were similar.

## 5. Conclusions

House dust mite is a highly prevalent domestic source and a significant cause of allergic diseases. It is urgent to find effective, harmless, and ecologically friendly materials to reduce the allergenicity of HDM allergens. According to our findings, todomatsu oil has been shown in vitro to inhibit the allergenicity of Der f 2, one kind of the most crucial HDM allergens from *Dermatophagoides farinae*, via decreasing its amount, instead of lowering the IgG binding ability of a single protein. Furthermore, the alkyl hydrophobic interactions have been observed between Der f 2 and chemical components in todomatsu oil. Notably, the majority of the compounds in todomatsu oil interact with the hydrophobic amino acids buried deep within the protein Der f 2, and here are seven compounds (β-myrcene, limonene, camphene, 3-carene, α-terpinolene, β-maaliene, and β-phellandrene) interact with the amino acid residue CYS27 which formed a disulfide bond with CYS21 in Der f 2. In contrast, the primary compounds in todomatsu oil fail to attach to the amino acids forming the IgG epitope. Surprisingly, compounds with the lowest relative percentages in todomatsu oil show the highest binding affinity values on Der f 2, particularly β-maaliene. Todomatsu oil, which is produced from the residues of *Abies Sachalinensis* and decreases the allergenicity of both cedar pollen and house dust mites, is effective, harmless, low-cost, and environmentally friendly instead of the more expensive medicinal plants. Utilizing the essential oil extracted from plant residues is a promising public health technique to prevent pollinosis and house dust mite allergy.

## Figures and Tables

**Table 1:** Pretreatment of samples for different analyses.

Samples	Essential Oil (μL/cm^3^)	Allergen Der f 2 (ng/mL)	Exposure Temperature (°C)	Exposure Time (h)
**Using for ELISA**	−	250 250 2000	20 25 30	1, 2, 4, 8
	0.05	250 250 2000	20 25 30	1, 2, 4, 8
**Using for SPR experiments**	−	500, 250, 125, 62.5, and 31.25	25	1
	0.05	500, 250, 125, 62.5, and 31.25	25	1
**Using for SDS-PAGE**	−	100,000	25	2
	0.05	100,000	25	2

“−”: without todomatsu oil. ELISA, enzyme-linked immunosorbent assay; SPR, surface plasmon resonance; SDS-PAGE, sodium dodecyl sulfate-polyacrylamide gel electrophoresis.

**Table 2:** Comparison of dissociation constants (K_D_) and maximum binding (Rmax).

Samples	K_D_ (M)		Rmax (RU)	
	1st Time	2nd Time	1st Time	2nd Time
Der f 2 without todomatsu oil	2.217 × 10^−9^	2.255 × 10^−9^	476.2	456.5
Der f 2 with todomatsu oil	2.221 × 10^−9^	2.110 × 10^−9^	460.5	442.6

Der f 2 (500, 250, 125, 62.5, and 31.25 ng/mL) were treated with and without todomatsu oil (0.05 µL/cm^3^) at 25 °C for 1 h. The single-cycle kinetics (SCK) method of surface plasmon resonance (SPR) was performed to determine the Ig G binding ability of a single protein Der f 2. Two independent replicates and the results are described as the 1st or 2nd time. K_D_ represents the binding strength of the antigen and its specific antibody. A lower K_D_ value indicates a higher binding capability. Rmax represents the maximum binding of the antigen on its specific antibody.

**Table 3:** Interactions of the dominant chemical compounds in todomatsu oil with amino acid residues on Der f 2 (PDB ID: 1WRF).

Chemical Compounds	PubChem CID	Chemical Formula	Chemical 3D Structure	Chemical 2D Structure	ΔG Energy (kcal/mol)	Protein–Ligand Interaction	Interacting Amino Acid Residues
(a) 3-carene	26049	C_10_H_16_	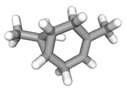	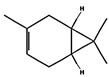	−7.1	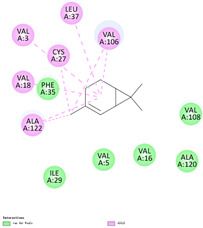	VAL3; VAL18; CYS27; LEU37; VAL106; ALA122
(b) α-terpinolene	11463	C_10_H_16_	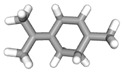	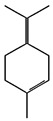	−7.2	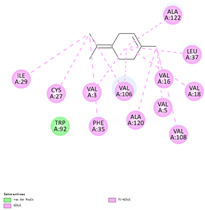	VAL3; VAL5; VAL16; VAL18; CYS27; ILE29; PHE35; LEU37; VAL106; VAL108; ALA120; ALA122
(c) borneol	64685	C_10_H_18_O	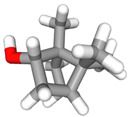	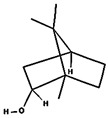	−5.2	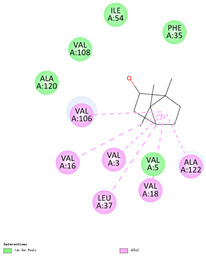	VAL3; VAL16; VAL18; LEU37; VAL106; ALA122
(d) β-maaliene	101596917	C_15_H_24_	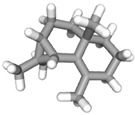	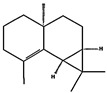	−8.0	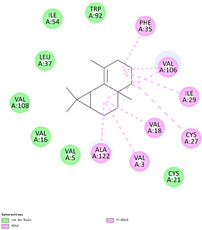	VAL3; VAL18; CYS27; ILE29; PHE35; VAL106; ALA122
(e) tricyclene	79035	C_10_H_16_	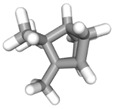	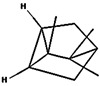	−6.5	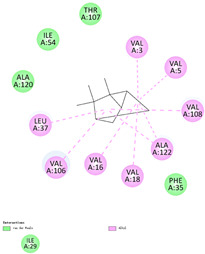	VAL3; VAL5; VAL16; VAL18; LEU37; VAL106; VAL108; ALA122
(f) β-myrcene	31253	C_10_H_16_	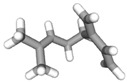	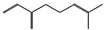	−6.3	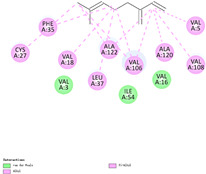	VAL5; VAL18; CYS27; PHE35; LEU37; VAL106; VAL108; ALA120; ALA122
(g) limonene	22311	C_10_H_16_	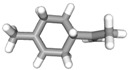	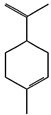	−6.8	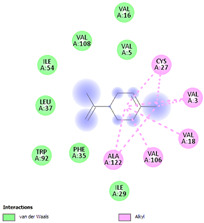	VAL3; VAL18; CYS27; VAL106; ALA122
(h) β-pinene	14896	C_10_H_16_	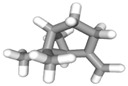	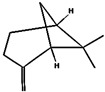	−6.8	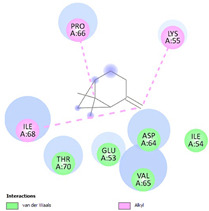	LYS55; PRO66; ILE68
(i) β-phellandrene	11142	C_10_H_16_	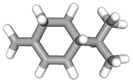	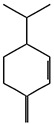	−7.0	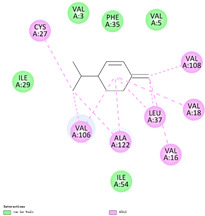	VAL16; VAL18; CYS27; LEU37; VAL106; VAL108; ALA122
(j) α-pinene	6654	C_10_H_16_	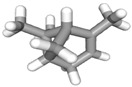	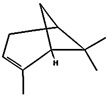	−6.6	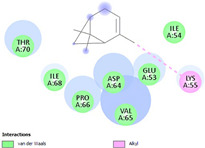	LYS55
(k) camphene	6616	C_10_H_16_	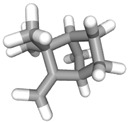	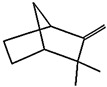	−6.5	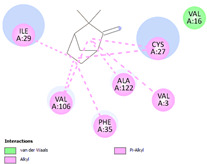	VAL3; CYS27; ILE29; PHE35; VAL106; ALA122
(l) bornyl acetate	6448	C_12_H_20_O_2_	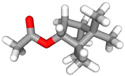	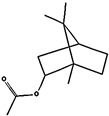	−6.2	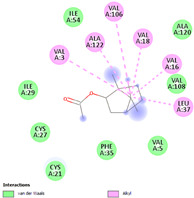	VAL3; VAL16; VAL18; LEU37; VAL106; ALA122

VAL: valine; CYS: cysteine; LEU: leucine; ALA: alanine; ILE: isoleucine; PHE: phenylalanine; LYS: lysine; PRO: proline.

## Data Availability

The raw data requests can be made to the corresponding author.

## References

[1] Zhang J., Tao A. (2015). Antigenicity, Immunogenicity, Allergenicity. Allergy Bioinform..

[2] Huang F.L., Liao E.C., Yu S.J. (2018). House Dust Mite Allergy: Its Innate Immune Response and Immunotherapy. Immunobiology.

[3] Meng Q., Liu X., Li P., He L., Xie J., Gao X., Wu X., Su F., Liang Y. (2016). The Influence of House Dust Mite Sublingual Immunotherapy on the TSLP-OX40L Signaling Pathway in Patients with Allergic Rhinitis. Int. Forum Allergy Rhinol..

[4] Platts-Mills T.A.E., Erwin E.A., Heymann P.W., Woodfolk J.A. (2009). Pro: The Evidence for a Causal Role of Dust Mites in Asthma. Am. J. Respir. Crit. Care Med..

[5] Dabbaghzadeh A., Ghaffari J., Feridoni M., Alipour A. (2020). Research Paper: House Dust Mite Allergen Levels of Der p 1 and Der f 1 in Houses of Asthmatic Children. J. Pediatr. Rev..

[6] Yasuda Y., Nagano T., Kobayashi K., Nishimura Y. (2020). Group 2 Innate Lymphoid Cells and the House Dust. Cells.

[7] Linneberg A., Hernandez D., De Rojas F., Virchow J.C., Demoly P., Kingdom U. (2015). Respiratory Allergy Caused by House Dust Mites: What Do We Really Know ?. J. Allergy Clin. Immunol..

[8] Ichikawa S., Takai T., Inoue T., Yuuki T., Okumura Y., Ogura K., Inagaki F., Hatanaka H. (2005). NMR Study on the Major Mite Allergen Der f 2: Its Refined Tertiary Structure, Epitopes for Monoclonal Antibodies and Characteristics Shared by ML Protein Group Members. J. Biochem..

[9] Inohara N., Nuez G. (2002). ML—A Conserved Domain Involved in Innate Immunity and Lipid Metabolism. Trends Biochem. Sci..

[10] Jeong K.Y., Lee I.Y., Ree H.I., Hong C.S., Yong T.S. (2002). Localization of Der f 2 in the Gut and Fecal Pellets of Dermatophagoides Farinae. Allergy.

[11] Johannessen B.R., Skov L.K., Kastrup J.S., Kristensen O., Bolwig C., Larsen J.N., Spangfort M., Lund K., Gajhede M. (2005). Structure of the House Dust Mite Allergen Der f 2: Implications for Function and Molecular Basis of IgE Cross-Reactivity. FEBS Lett..

[12] Cao H., Liu Z. (2020). Clinical Significance of Dust Mite Allergens. Mol. Biol. Rep..

[13] Thomas W.R., Hales B.J. (2007). T and B Cell Responses to HDM Allergens and Antigens. Immunol. Res..

[14] Yang L., Hirose S., Suzuki K., Hiroi T., Takaiwa F. (2012). Expression of Hypoallergenic Der f 2 Derivatives with Altered Intramolecular Disulphide Bonds Induces the Formation of Novel ER-Derived Protein Bodies in Transgenic Rice Seeds. J. Exp. Bot..

[15] Nishiyama C., Yuuki T., Takai T., Okumura Y., Okudaira H. (1993). Determination of Three Disulfide Bonds in a Major House Dust Mite Allergen, Der f II. Int. Arch. Allergy Immunol..

[16] Jung J. (2021). Insecticidal Effect against House Dust Mite Using Ethanol Extract of *Theobroma cacao* L.. Ann. Rom. Soc. Cell Biol..

[17] Abidin S.Z., Ming H.T. (2012). Effect of a Commercial Air Ionizer on Dust Mites Dermatophagoides Pteronyssinus and Dermatophagoides Farinae (Acari: Pyroglyphidae) in the Laboratory. Asian Pac. J. Trop. Biomed..

[18] Murray C.S., Foden P., Sumner H., Shepley E., Custovic A., Simpson A. (2017). Preventing Severe Asthma Exacerbations in Children a Randomized Trial of Mite-Impermeable Bedcovers. Am. J. Respir. Crit. Care Med..

[19] Halken S., Høst A., Niklassen U., Hansen L.G., Nielsen F., Pedersen S., Østerballe O., Veggerby C., Poulsen L.K. (2003). Effect of Mattress and Pillow Encasings on Children with Asthma and House Dust Mite Allergy. J. Allergy Clin. Immunol..

[20] Terreehorst I., Hak E., Oosting A.J., Tempels-Pavlica Z., de Monchy J.G.R., Bruijnzeel-Koomen C.A.F.M., Aalberse R.C., van Wijk R.G. (2003). Evaluation of Impermeable Covers for Bedding in Patients with Allergic Rhinitis. N. Engl. J. Med..

[21] Van Den Bemt L., Van Knapen L., De Vries M.P., Jansen M., Cloosterman S., Van Schayck C.P. (2004). Clinical Effectiveness of a Mite Allergen-Impermeable Bed-Covering System in Asthmatic Mite-Sensitive Patients. J. Allergy Clin. Immunol..

[22] Tsurikisawa N., Saito A., Oshikata C., Nakazawa T., Yasueda H., Akiyama K. (2013). Encasing Bedding in Covers Made of Microfine Fibers Reduces Exposure to House Mite Allergens and Improves Disease Management in Adult Atopic Asthmatics. Allergy Asthma Clin. Immunol..

[23] Rijssenbeek-Nouwens L.H.M., Oosting A.J., De Bruin-Weller M.S., Bregman I., De Monchy J.G.R., Postma D.S. (2002). Clinical Evaluation of the Effect of Anti-Allergic Mattress Covers in Patients with Moderate to Severe Asthma and House Dust Mite Allergy: A Randomised Double Blind Placebo Controlled Study. Thorax.

[24] Gore R.B., Durrell B., Bishop S., Curbishley L., Woodcock A., Custovic A. (2006). High-Efficiency Vacuum Cleaners Increase Personal Mite Allergen Exposure, but Only Slightly. Allergy Eur. J. Allergy Clin. Immunol..

[25] Maloney J., Sicherer S.H. (2005). Results of a Home-Based Environmental Intervention among Urban Children with Asthma. Pediatrics.

[26] Antonicelli L., Bilò M.B., Pucci S., Schou C., Bonifazi F. (1991). Efficacy of an Air-cleaning Device Equipped with a High Efficiency Particulate Air Filter in House Dust Mite Respiratory Allergy. Allergy.

[27] Reisman R.E., Mauriello P.M., Davis G.B., Georgitis J.W., DeMasi J.M. (1990). A Double-Blind Study of the Effectiveness of a High-Efficiency Particulate Air (HEPA) Filter in the Treatment of Patients with Perennial Allergic Rhinitis and Asthma. J. Allergy Clin. Immunol..

[28] Arlian L.G., Neal J.S., Vyszenski-Moher D.A.L. (1999). Reducing Relative Humidity to Control the House Dust Mite Dermatophagoides Farinae. J. Allergy Clin. Immunol..

[29] Hayden M.L., Rose G., Diduch K.B., Domson P., Chapman M.D., Heymann P.W., Platts-Mills T.A.E. (1992). Benzyl Benzoate Moist Powder: Investigation of Acarical Activity in Cultures and Reduction of Dust Mite Allergens in Carpets. J. Allergy Clin. Immunol..

[30] Lee I., Park J. (2007). Insecticidal Effect of Dermatoohagoides Pteronyssinus Using Ginkgo Biloba Leaves Extracts. KSBB J..

[31] Hayes W.J., Laws E.R. (1991). Handbook of Pesticide Toxicology.

[32] Doungnapa T., Pumnuan J., Insung A. (2021). Acaricidal Activity of Essential Oil Nanoemulsion against the African Red Mite (Eutetranychus Africanus). Chil. J. Agric. Res..

[33] Huang C.X., Li H.G., Luo H.Q., Fu Q.M., He B.S., Bao M.H. (2021). Essential Oils for the Treatment of Demodex. E3S Web Conf..

[34] Yu H., Ren X., Yang F., Xie Y., Guo Y., Cheng Y., Yao W. (2021). Antimicrobial and Anti-Dust Mite Efficacy of Cinnamomum Camphora Chvar. Borneol Essential Oil Using Pilot-Plant Neutral Cellulase-Assisted Steam Distillation. Lett. Appl. Microbiol..

[35] Bogdan M.A., Bungau S., Tit D.M., Zaha D.C., Nechifor A.C., Behl T., Chambre D., Lupitu A.I., Copolovici L., Copolovici D.M. (2021). Chemical Profile, Antioxidant Capacity, and Antimicrobial Activity of Essential Oils Extracted from Three Different Varieties (Moldoveanca 4, Vis Magic 10, and Alba 7) of Lavandula Angustifolia. Molecules.

[36] Insung A., Pumnuan J., Mahakittikun V., Wangapai T. (2016). Effectiveness of Essential Oils of Medicinal Plants at Reducing the Amounts of Allergen Produced by the European House Dust Mite, *Dermatophagoides Pteronyssinus* (Trouessart). J. Acarol. Soc. Jpn..

[37] Tatsuro O., Naoyuki M., Toshihiko K., Yuichi T.M. (2010). Efficient extraction of essential oil from woody materials using vaccume microwave assisted steam distillation. Aroma Res..

[38] STC 2013 The Air Purifier “Clear Forest” Is Born!—Abies Sachalinensis Oil Cleans Odors and Dirt *! -New Release of 2 Types for Cars for Air Conditioner Louvers/for Under-Seat and Side Pockets. https://www.st-c.co.jp/news/newsrelease/2013/20131003_002911.html.

[39] Nishiyama C., Hatanaka H., Ichikawa S., Fukada M., Akagawa-Chihara M., Yuuki T., Yokota T., Inagaki F., Okumura Y. (1999). Analysis of Human IgE Epitope of Der f 2 with Anti-Der f 2 Mouse Monoclonal Antibodies. Mol. Immunol..

[40] Dallakyan S., Olson A.J. (2015). Small-Molecule Library Screening by Docking with PyRx. Methods Mol. Biol..

[41] Duru I.A., Duru C.E., Enyoh C.E., Umar H.I. (2022). Computer-Aided Degradation Susceptibility Study of Crude Oil Compounds at Bacillus Subtilis Protein Target. Environ. Eng. Res..

[42] Cabanillas B., Pedrosa M.M., Rodríguez J., González Á., Muzquiz M., Cuadrado C., Crespo J.F., Burbano C. (2010). Effects of Enzymatic Hydrolysis on Lentil Allergenicity. Mol. Nutr. Food Res..

[43] Lv L., Qu X., Yang N., Ahmed I. (2021). The Conformational Structural Change of β-Lactoglobulin via Acrolein Treatment Reduced the Allergenicity. Food Chem. X.

[44] Zhou S., Zhao H., Peng J., Hong Q., Xiao K., Shang Y., Lu S., Zhang W., Wu M., Li S. (2019). Size Distribution of Platanus Acerifolia Allergen 3 (Pla A3) in Shanghai Ambient Size-Resolved Particles and Its Allergenic Effects. Atmos. Environ..

[45] He W., He K., Sun F., Mu L., Liao S., Li Q., Yi J., Liu Z., Wu X. (2021). Effect of Heat, Enzymatic Hydrolysis and Acid-Alkali Treatment on the Allergenicity of Silkworm Pupa Protein Extract. Food Chem..

[46] Pi X., Fu G., Dong B., Yang Y., Wan Y., Xie M. (2021). Effects of Fermentation with Bacillus Natto on the Allergenicity of Peanut. Lwt.

[47] Visentin J., Couzi L., Dromer C., Neau-Cransac M., Guidicelli G., Veniard V., Coniat K.N.L., Merville P., Di Primo C., Taupin J.L. (2018). Overcoming Non-Specific Binding to Measure the Active Concentration and Kinetics of Serum Anti-HLA Antibodies by Surface Plasmon Resonance. Biosens. Bioelectron..

[48] Visentin J., Minder L., Lee J.H., Taupin J.L., Di Primo C. (2016). Calibration Free Concentration Analysis by Surface Plasmon Resonance in a Capture Mode. Talanta.

[49] Trott O., Olson A.J. (2009). AutoDock Vina: Improving the Speed and Accuracy of Docking with a New Scoring Function, Efficient Optimization, and Multithreading. J. Comput. Chem..

[50] Rappé A.K., Casewit C.J., Colwell K.S., Goddard W.A., Skiff W.M. (1992). UFF, a Full Periodic Table Force Field for Molecular Mechanics and Molecular Dynamics Simulations. J. Am. Chem. Soc..

[51] O’Boyle N.M., Banck M., James C.A., Morley C., Vandermeersch T., Hutchison G.R. (2011). Open Babel: An open chemical toolbox. J. Cheminform..

[52] Duru C.E., Umar H.I.U., Duru I.A., Enenebeaku U.E., Ngozi-Olehi L.C., Enyoh C.E. (2021). Blocking the Interactions between Human Ace2 and Coronavirus Spike Glycoprotein by Selected Drugs: A Computational Perspective. Environ. Health Toxicol..

[53] Duru C.E., Duru I.A., Enyoh C.E. (2021). In Silico Binding Affinity Analysis of Microplastic Compounds on PET Hydrolase Enzyme Target of Ideonella Sakaiensis. Bull. Natl. Res. Cent..

[54] Li J., Hu Y., Li H., Lin Y., Tong S., Li Y. (2022). Assessing the Impact of Air Pollutants on Clinical Visits for Childhood Allergic Respiratory Disease Induced by House Dust Mite in Shanghai, China. Respir. Res..

[55] Yucel E., Suleyman A., Demirkale Z.H., Guler N., Tamay Z.U., Ozdemir C. (2021). ‘Stay at Home’: Is It Good or Not for House Dust Mite Sensitized Children with Respiratory Allergies?. Pediatr. Allergy Immunol..

[56] Takai T., Ichikawa S., Yokota T., Hatanaka H., Inagaki F., Okumura Y. (2000). Unlocking the Allergenic Structure of the Major House Dust Mite Allergen Der f 2 by Elimination of Key Intramolecular Interactions. FEBS Lett..

[57] STC 2018 New Brand of Health Care Products “MoriLabo” New Release of “MoriLabo Pollen Barrier Stick”, Which is a Scent to Prevent Pollen just by Applying It to the Mask. https://www.st-c.co.jp/news/newsrelease/2018/20181128_004349.html.

[58] Jakobsen C.G., Bodtger U., Poulsen L.K., Roggen E.L. (2005). Vaccination for Birch Pollen Allergy: Comparison of the Affinities of Specific Immunoglobulins E, G1 and G4 Measured by Surface Plasmon Resonance. Clin. Exp. Allergy.

